# Grazer exclusion alters plant spatial organization at multiple scales, increasing diversity

**DOI:** 10.1002/ece3.743

**Published:** 2013-09-02

**Authors:** Hui Zhang, Benjamin Gilbert, Wenbin Wang, Junjie Liu, Shurong Zhou

**Affiliations:** 1 State Key Laboratory of Grassland Agro-ecosystems, School of Life Sciences, Lanzhou UniversityLanzhou, 730000, China; 2 Department of Ecology and Evolutionary Biology, University of Toronto25 Harbord St., Toronto, Ontario, M5S 3G5, Canada; 3 Ministry of Education Key Laboratory for Biodiversity Science and Ecological Engineering, School of Life Sciences, Fudan University220 Handan Road, Shanghai, 200433, China

**Keywords:** Biodiversity, community, interspecific association, intraspecific association, meadow, spatial scale, species richness

## Abstract

Grazing is one of the most important factors influencing community structure and productivity in natural grasslands. Understanding why and how grazing pressure changes species diversity is essential for the preservation and restoration of biodiversity in grasslands. We use heavily grazed subalpine meadows in the Qinghai-Tibetan Plateau to test the hypothesis that grazer exclusion alters plant diversity by changing inter- and intraspecific species distributions. Using recently developed spatial analyses combined with detailed ramet mapping of entire plant communities (91 species), we show striking differences between grazed and fenced areas that emerged at scales of just one meter. Species richness was similar at very small scales (0.0625 m^2^), but at larger scales diversity in grazed areas fell below 75% of corresponding fenced areas. These differences were explained by differences in spatial distributions; intra- and interspecific associations changed from aggregated at small scales to overdispersed in the fenced plots, but were consistently aggregated in the grazed ones. We conclude that grazing enhanced inter- and intraspecific aggregations and maintained high diversity at small scales, but caused decreased turnover in species at larger scales, resulting in lower species richness. Our study provides strong support to the theoretical prediction that inter- and intraspecific aggregation produces local spatial patterns that scale-up to affect species diversity in a community. It also demonstrates that the impacts of grazing can manifest through this mechanism, lowering diversity by reducing spatial turnover in species. Finally, it highlights the ecological and physiological plant processes that are likely responding to grazing and thereby altering aggregation patterns, providing new insights for monitoring, and mediating the impacts of grazing.

## Introduction

Community composition and diversity are spatial phenomena determined by environmental heterogeneity and by spatially structured ecological processes such as disturbance, species interactions, and dispersal (Bennie et al. 2011). There is a growing awareness that the spatial distributions of plant species can provide important clues to critical determinants of biodiversity (Lang et al. 2009; Bennie et al. 2011). For example, it is well understood that the relative strengths of intra- and interspecific interactions underpin patterns of species diversity (Chesson 2000). When these processes manifest in spatial patterns of inter- and intraspecific clustering (e.g., Condit et al. 2000; Comita et al. 2010), they produce emergent ecological properties such as the slope of species–area curves (He and Legendre 2002). Despite this potential for spatial distributions to inform emergent patterns in community ecology (e.g., Condit et al. 2000; Comita et al. 2010), recently developed techniques have not yet to be used to test how different ecological processes, such as herbivory, scale up to produce community-level patterns.

Recently, a number of spatial statistical methods have been employed to understand the effects of ecological processes on communities (e.g., Condit et al. 2000; Zhang et al. 2012, 2013). These approaches are particularly interesting in areas that experience grazing because the net effect of herbivory on plant communities may be expressed by shifts in vegetation heterogeneity (Cyr and Face 1993; Deléglise et al. 2011). Herbivory is one of the most important components of land use that affects the structure of plant communities (Deléglise et al. 2011; Peper et al. 2011). Herbivores can directly shape plant communities through selective foraging, causing changes in species richness (Busso et al. 2004; Peper et al. 2011), functional group diversity (Rusch and Oesterheld 1997; Pokorny et al. 2005), and community productivity (Cyr and Face 1993; Post and Pedersen 2008). However, studies to date have shown varying effects of herbivory on the spatial heterogeneity of plant communities (e.g., Oliff and Ritchle 1998; Condit et al. 2000; Graff et al. 2007). Nonetheless, many of these studies have also been fairly limited in the number of species considered, and comparison across studies may therefore confound individualistic responses of different species with systemic differences in the effects of grazing. Advancement in this area requires that the effects of herbivory be measured across all species in a community and that the spatial methods used be comparable among diverse species.

Point process methods provide a powerful tool for examining how species' spatial distributions vary among communities, and how the outcomes of these differences scale-up to influence species diversity (Condit et al. 2000; Shimatani and Kubota 2004). For example, Condit et al. (2000) used this approach to test spatial aggregation of tropical trees, and found that patterns of trees are inconsistent with the Janzen–Connell hypothesis. Just as the Condit approach is a powerful measure of intraspecific aggregation, bivariate point process methods can be used to explore interspecific association (or conversely, overdispersion) for any number of species pairs (Wang et al. 2010). These two approaches, when coupled, correspond with the patterns produced by intra- and interspecific interactions, which together are sufficient to explain species diversity in many models of interacting species (Chesson 2000; He and Legendre 2002).

In this article, we use point process methods to understand how exclusion of grazers affects inter- and intraspecific associations, and how changing these associations impacts species diversity. We study grasslands in Qinghai-Tibetan Plateau meadows that were heavily grazed for over 30 years prior to being divided into fenced (grazers excluded) or unfenced (grazing maintained) areas in 2000. This area is of particular interest because overgrazing is considered widespread and has degraded the diversity and function of many grasslands (Lü et al. 2011). We established 10 × 10 m plots and mapped aboveground ramets of all species in all 0.05 × 0.05 m subquadrats. We contrast grazed and fenced plots to address the following questions: (1) How does the exclusion of grazers affect species diversity in this area? and (2) Are changes in diversity concordant with changes in intraspecific and interspecific associations observed in overgrazed and grazer-excluded meadows? On the basis of previous research that often shows a decrease in diversity with overgrazing (Lü et al. 2011), we hypothesize that this pattern would manifest through higher intraspecific aggregation within and less negative associations among species in the overgrazed area – these hypotheses correspond to lower intraspecific limitation and lower species turnover in overgrazed areas (He and Legendre 2002).

## Materials and Methods

### Study site

Field sampling was conducted in two species-rich subalpine meadows located in the eastern part of the Qinghai-Tibetan Plateau, Hezuo, China (34°55**'**N, 102°53**'**E; 2900 m a.s.l.). Mean annual precipitation of 530 mm is mainly distributed in summer and the mean annual temperature is 2.4^°^. The vegetation at the study sites is typical of species-rich subalpine meadows, which is dominated by *Elymus nutans* Griseb, *Kobresia humilis* (*C.A. Mey*.) *serg.,* and *Thermopsis lanceolate R. Br*. Soils are classified as alpine meadow soils (Zhang et al. 2012, 2013), and soil resource characteristics are given in Table S1.

The focal meadows are in a large area of 4000 ha. The area studied was divided into two parts by fencing to prevent grazing in 2000. Hence, “grazed” areas have been overgrazed by livestock (mainly yaks and sheep) year round for more than 30 years and “fenced” areas have been protected from grazing since 2000. One 10 × 10 m plot was arranged in 2011 in a fenced area and an adjacent overgrazed area of one meadow, with a 10 m spacing between the closest edges of the two plots. We replicated the study in 2012 at a meadow 5 km away from the first one. All plots had similar orientation, aspect, and slope position.

Field investigation was performed in August during the peak of the growing season. We divided each plot into 100 quadrats of size 1 × 1 m by thin wires. Sampling was conducted at the scale of 0.05 × 0.05 m subquadrats within each quadrat providing very precise spatial locations for all aboveground ramets. We collected occurrence data for all species in the subquadrats to approximate the *x*- and *y*-coordinate of each ramet of every species. This is an approximation of ramet mapping in the 10 × 10 m plots. Given that the diameter of the base of species varies from ∼0.01 to 0.04 m, 0.05 × 0.05 m quadrats gave the best spatial resolution possible. In order to quantify habitat heterogeneity, we selected 50 points in each plot (distributed at regular intervals) and measured slope and several soil variables that are considered important for plants: total nitrogen, carbon, phosphorus, potassium, calcium, sodium, magnesium, iron, soil water content, and PH.

### Statistical analysis

To compare species richness in the fenced and the grazed plots at different scales, we first constructed nested species–area curves. Species–area curves were generated by calculating average richness in nonoverlapping, regularly arranged quadrats of a given size, with quadrat size varied by sampling quadrats of different sizes from the ramet maps of the plots. We selected 11 quadrat sizes: 0.25 × 0.25 m (1600 quadrats were sampled), 0.25 × 0.5 m (800), 0.5 × 0.5 m (400), 0.5 × 1 m (200), 1 × 1 m (100), 1 × 2 m (50), 2 × 2 m (25), 2 × 4 m (10), 4 × 4 m (4), 4 × 8 m (2), and 8 × 8 m (1).

#### Univariate spatial point patterns

Second order point pattern analyses are the most widely used methods to analyze the spatial distribution of mapped point data. They include Ripley's *K* function (Ripley 1977) and the pair correlation g function (Stoyan and Penttinen 2000). Ripley's *K* function is cumulative, which may confound large scale (large distance) effects with effects of small scales. The *g*-function, which we use, describes how Ripley's *K* changes with spatial distance and can thus separate scale effects (Diggle 2003). Because points close to plot edges will have less observations within the sampling circle than points at the plot center, we use the weighted unbiased estimator of *K*(*r*) (Ripley 1977) to compute the *g* function. A *g* function value greater than one indicates aggregation, whereas less than one indicates a regular (overdispersed) distribution.

In order to compare measures of aggregation among species with different densities, we use a measure that standardizes aggregation relative to a species' overall density. In particular, we use *g*_0–0.5_, the mean conspecific density within 0.5 m of a ramet relative to the species' overall density, as a simple measure of a species' aggregation intensity. The *g*_0–0.5_ index has been scaled by the relative density of the species and can therefore be used for comparison among different species (Condit et al. 2000). We chose the distance of 0.5 m to standardize this measure because it represented a distance that was small enough for most species to be aggregated, but large enough to capture a reasonable number of ramets. The number of aboveground ramets for several species are too numerous at large plot radii to estimate *g*(*r*). Hence, we restricted the *r* values to a maximum of 1/4 of the length of the sampling area. We used 99 Monte Carlo simulations of point processes generated by a heterogeneous Poisson process to test the hypothesis that a species was not significantly different from a random distribution (*g* = 1).

In order to test associations below the 0.5 m scale, we used a null model that randomizes the data conditionally on the observed larger-scale pattern. In practice, this was done by displacing the known locations of ramets randomly within a neighborhood within radius *R* (Wiegand et al. 2007; Wang et al. 2010). This displacement removes potential patterns for distances less than *R*, but it leaves the larger-scale patterns untouched. Contrasting the observed pattern to randomizations from this null model therefore detects only smaller-scale effects, and thus provides a test that is independent from *g*(*r*) at larger scales. Ninety-nine distributions were simulated by randomly labeling all the aboveground ramets within radius *R* (Wiegand et al. 2007; Wang et al. 2010), while keeping the abundance of each species the same as the observed. For all tests, if the observed value fell outside the 2.5th or 97.5th quartiles of the null model, we concluded that the focal species was significantly aggregated or regularly distributed (overdispersion). To detect the relationship between species abundances and aggregation, we conducted a regression analysis of *g*_0–0.5_ against the abundance for all species in the plots.

#### The bivariate spatial point patterns

The pair correlation g function can be extended to bivariate pair correlation function *g*_12_(*r*) to quantify species associations. Detailed descriptions of this function can be found in (Wiegand and Moloney 2004; Wang et al. 2010). We used the weighted unbiased estimator of *K*(*r*) to compute *g*_12_(*r*). We again conducted 99 Monte Carlo simulations to test whether species pairs in the two plots were significantly positively associated (attraction, *g*_12_(*r*) >1), negatively associated (repulsion, *g*_12_(*r*) <1), or indistinguishable from random (*g*_12_(*r*) = 1).We selected a maximum distance of *R* = 2.5 m to compute *g*_12_(*r*) because of the edge effects and the limited range of neighborhood effects (Wiegand et al. 2007; Wang et al. 2010).

## Results

**Figure 1:**
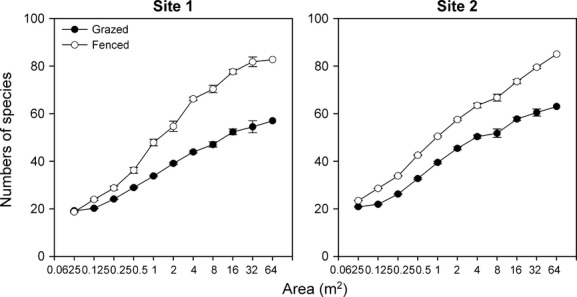
Species–area curves in fenced and grazed areas. Curves are nested so that values at large areas contain those from smaller areas within a given site and treatment. Each point represents the mean and standard error of the number of species found in a given area of the control and grazed plots.

The 160,000 sampled subplots (0.05 × 0.05 m) contained 91 herbaceous species (including graminoids and herbs species) in total. Eighty-four of these species occurred in the fenced plot and 61 in the grazed plot in site 1, and 85 in the fenced plot and 63 in the grazed plot in site 2 (see Table S2). At small spatial scales of 0.0625 m^2^, species richness was similar between two grazed (19.24 ± 0.07 SE and 21.91 ± 0.28 SE) and two fenced (18.75 ± 0.12 SE and 23.48 ± 0.09 SE) plots, but the fenced plots gained relatively more species as the spatial scale of sampling increased (Fig. 1). This difference in richness between grazed and fenced plots at large scales relative to small scales indicates a higher degree of species turnover at large scales in the fenced area.

Patterns of species aggregation may be influenced directly through species interactions or indirectly through grazing-mediated changes to local conditions. Our analysis of soil heterogeneity indicated that the maximum variation in soil resources appeared to be influenced by our treatment, as grazed areas had slightly higher mean and higher coefficient of variation in soil N and P (higher sills in the variograms, Figs. S1, S2; Table S1). These differences suggest that one impact of grazing may be indirect through soil resources. It is also important to note that the minimum distance of soil resource measurement was 1.4 m, which is larger than the scale at which patterns of aggregation began to diverge in the grazed and fenced areas. As a result, it is possible that grazer exclusion altered the soil environment at very fine scales that we were unable to detect. Because of this uncertainty, we only consider the effect of soil variation at the larger scales (≥1.4 m) at which it was measured.

**Figure 2:**
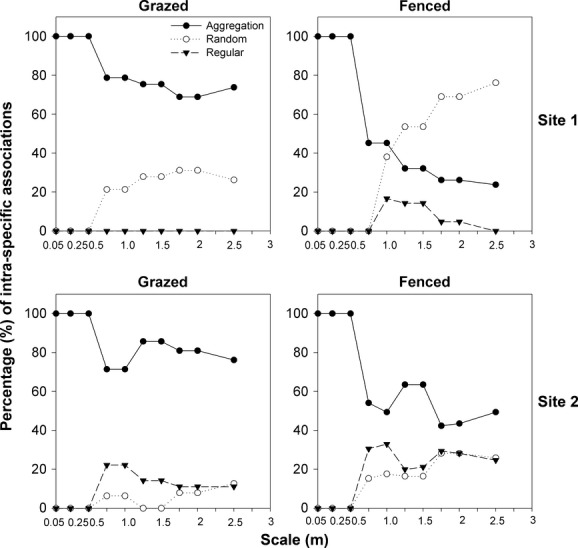
Patterns of intraspecific association across spatial scales based on the function *g*(*r*). Associations were considered significantly nonrandom if they fell outside the 95% confidence intervals of the null model (see Material and Methods).

In both the fenced and grazed plots of the two sites, all species showed significant intraspecific aggregation over the spatial scale of 0–0.5 m (Fig. 2). Intraspecific aggregation was almost uniformly high in grazed plots at all scales, whereas fenced plots showed large decreases in the amount of intraspecific aggregation beyond 0.5 m. Several species showed overdispersion (Fig. 2) in fenced plots, but there was no consistent pattern of overdispersion in grazed plots. In other words, although all the species in either grazed and fenced plots showed intraspecific aggregation at the scale of 0–0.5 m, the proportion of aggregated species was always higher in the grazed plots than the fenced plots with increasing spatial scales. In contrast, the number of species that were randomly distributed or overdispersed was higher in the two fenced plots. Overgrazing also caused some species to become more aggregated or enlarged the scale of aggregation, as is illustrated for three common species (Fig. S3).

**Figure 3:**
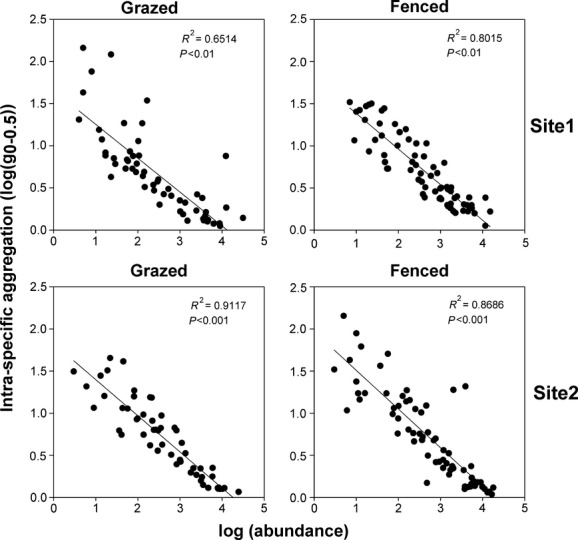
The relationship between intraspecific species aggregation (*g*_0–0.5_) and species abundances.

In grazed plots, the degree of intraspecific aggregation was greatest in low-abundance species and weakest in high-abundance species (Fig. 3). An identical pattern emerged in fenced plots, indicating that intraspecific aggregation was negatively associated with abundance regardless of the effects of grazing at this small scale (Fig. 3).

**Figure 4:**
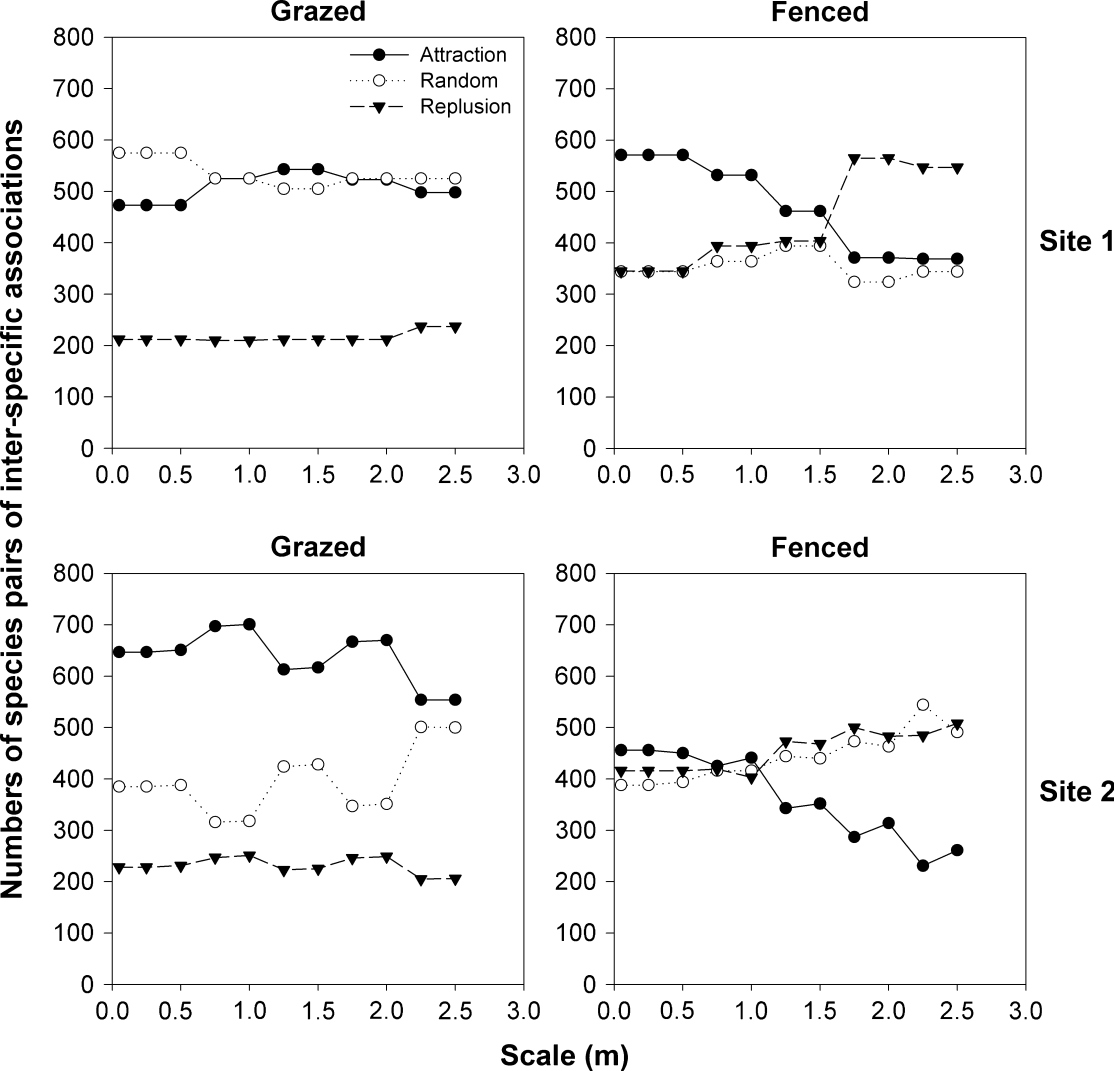
Patterns of interspecific association across spatial scales based on the function *g*_12_(*r*).Associations were considered significantly nonrandom if they fell outside the 95% confidence intervals of the null model (see Material and Methods).

To measure interspecific aggregation, we included 36 abundant species found in both grazed and fenced areas in a bivariate spatial point analysis. For these 36 species, there were a total of 36 × 35 = 1260 bivariate spatial point analyses in each plot type. The number of species pairs that were negatively associated (repulsion) was always larger in fenced plots than in grazed plots (Fig. 4). Overall, less species were positively associated (attraction) in the fenced than in the grazed plots – grazed areas maintained positive species associations, whereas many species associations shifted from positive to negative in the fenced plots (Fig. S4). Negative species associations were predominant at larger scales in the fenced plots, whereas positive or random associations were predominant at all scales in grazed plots. These interspecific patterns mirrored patterns of intraspecific aggregation, which were aggregated at all scales in grazed plots, but only at very small scales in fenced plots.

## Discussion

Our study shows how spatial patterns of inter- and intraspecific aggregation influence species diversity from small to large scales (0.06–64 m^2^) in a community. This scaling, predicted by theoretical models (He and Legendre 2002), proves to be robust in heavily grazed grasslands that differ in whether they continue to be grazed or have large herbivores excluded. To our knowledge, our study is the first to perform these analyses for the majority of species in a community, and provides an important new tool for understanding the links between small-scale spatial distributions and the emergent properties of communities.

Theories of species diversity predict that strong intraspecific density dependence leads to higher diversity, and that one mechanism inducing intraspecific density dependence is spatial aggregation of conspecifics (Chesson 2000). Our results are partially consistent with this hypothesis, with rare species tending to be much more spatially aggregated at small scales (Fig. 3). A similar pattern has been observed in tropical forests (Condit et al. 2000), suggesting that it could be common to many ecosystems and may provide insight into the types of rarity found in many plant communities (Rabinowitz 1981). The negative relationship between abundance and aggregation also suggests that spatially mediated negative density dependence is important in this ecosystem, as has been shown in tropical forests (e.g., Comita et al. 2010). Although we do not test the underlying mechanisms for this pattern here, they may range from specialist insect herbivores (Janzen–Connell effects [Condit et al. 2000]), to species-specific responses to environmental conditions (Bagchi et al. 2011).

Grazed and fenced areas diverged in intraspecific aggregation at all but the smallest spatial scales (Figs. 2, S3). Greater levels of intraspecific aggregation at large scales are consistent with the decrease in species richness observed in grazed areas (He and Legendre 2002), and suggests that the factors that limit species aggregations play an important role when grazers are excluded. Although several processes may cause these differences in plant spatial patterns, we propose that three are plausible. First, herbivores can directly reduce the spatial extension of some species through selective grazing and indirectly may favor the spatial homogeneity of less palatable species by locally decreasing their competitors (Adler et al. 2001; Pazos et al. 2007). Indeed, grazing did exclude many competitive species in the grass family (Table S2). Second, the greater intraspecific aggregation in the grazed areas may result from increased intraspecific facilitation, which has been shown to be important for recruitment in stressful environments (Fajardo and McIntire 2011). Third, grazing may have altered plant reproductive dynamics. For example, it has been proposed that grazing may favor species that spread via vegetative reproduction, thereby creating larger patches of conspecifics (Hulme 1996). In grasslands dominated by perennial species, trampling and defoliation can also influence the spatial extension and aggregation of clones by reducing clonal mobility (Bullock et al. 1994; Tamm et al. 2001), decreasing distance of lateral spread (Smit et al. 2010; Benot et al. 2011), through limitation of internode length (Amiaud et al. 2008) or fragmentation of clone patches (Charpentier et al. 1998). In addition, grazing may favor flowering and seed production in shorter stature plants, which would also limit seed dispersal (Thomson et al. 2011). Overall, these effects of grazing on reproductive dynamics may favor a smaller subset of species with specific life history characteristics that lead to higher intraspecific aggregation.

Just as intraspecific aggregation was higher in the grazed area at all but the smallest scales, the grazed area showed greater levels of nonnegative (positive or random) species associations at the same scales. This lack of negative interspecific associations at greater distances is equivalent to a lower level of species turnover, and thus a flatter slope in the species–area curve at greater distances (Fig. 1). Previous research and conceptual models have shown that interspecific interactions may shift from competitive to facilitative dynamics as an environment becomes more stressful (Tewksbury and Lloyd 2001; Bruno et al. 2003; Graff et al. 2007; Bagousse-Pinguet et al. 2011). The consistent pattern of positive species associations in grazed plots may be indicative of such a shift to facilitation, as has been shown in grazed areas when neighboring species act as biotic refuges (through physical barriers such as spines) or provide associational avoidance (Graff et al. 2007; Chu et al. 2009; Bagousse-Pinguet et al. 2011). In addition, grazed plots housed more short stature plants, and the associations observed may be due to the relationship between plant height and light availability. Reduced light competition among short stature plants may prevent some negative species associations that would otherwise occur from shading by tall-stature plants (Evju et al. 2009; Chu et al. 2010).

Patterns of spatial aggregation and diversity showed a surprising consistency across sites and treatments (Figs. 4), and also across spatial scales when comparing treatments (Figs. 2, 4). The consistency across spatial scales is particularly interesting in that it is inconsistent with findings from previous research (Chaneton and Facelli 1991; Oliff and Ritchle 1998). For example, Chaneton and Facelli (1991) found that herbivore exclusion decreased species richness at the scales used in our study, but that this change in richness was scale dependent. Although we cannot compare to their largest scales, we do show a consistent, positive effect of grazer exclusion on species richness across all but the smallest scales considered (Fig. 1). Similarly, Oliff and Ritchle (1998) predicted that large herbivores should create higher habitat heterogeneity, thus promoting diversity. Our analysis on soil resources showed that grazed plots did have higher variation in soil P and N at larger (≥1.4 m) scales, but that this did not correspond to higher plant diversity, suggesting that direct negative effects of heavy grazing is more important than its indirect effects. Overall, these different results likely highlight the importance of grazing pressure (Lü et al. 2011), where overgrazing may cause patterns opposite to those seen at natural levels of grazing (Oliff and Ritchle 1998).

In summary, we have shown how patterns of species' intra- and interspecific associations can differ among overgrazed meadows and meadows excluded from grazing, and can scale-up to the total species richness of an area. This has previously been predicted by theory and simulation studies (He and Legendre 2002), but to our knowledge, we are the first to show this by contrasting different communities. Our study not only links small-scale patterns to emergent community properties but also highlights the processes that are likely responsible for these patterns, providing a way forward for understanding how the impacts of grazing could be monitored or mediated. In particular, linking local species distributions to emergent patterns may elucidate some of the varying effects of grazing on species diversity (Proulx and Mazumder 1998). Our study provides an important step in this direction.

## Supporting Information

**Table S1.** The average content of soil and topographic variables and the standard deviations (in brackets) in the fenced and grazed plots of site 1 and site 2.

**Table S2.** Species composition in the 10 × 10 m plots of the fenced and grazed areas of two meadows. The cross indicates that the species was detected in the plot.

**Figure S1.** Changes in resource heterogeneity as a function of distance between sampling points in site 1. Each point represents the average coefficient of variation for all pairwise points measured at that distance, with filled circles representing the CV in grazed areas and open circles representing the CV in fenced plots. Labels above each panel indicate the soil resource in site 1.

**Figure S2.** Changes in resource heterogeneity as a function of distance between sampling points in site 2. Each point represents the average coefficient of variation for all pairwise points measured at that distance, with filled circles representing the CV in grazed areas and open circles representing the CV in fenced plots. Labels above each panel indicate the soil resource in site 2.

**Figure S3.** The examples of the intraspecific spatial patterns (univariate point process) of the three types of species (dominating, common and rare species) in the fenced and grazed plots. The solid lines are for the values of g function and the two dotted lines represent the upper and the lower limits of the 95% confidence envelopes of the 99 Monte Carlo simulations by randomly labeling all the aboveground-ramets in the plot while keeping the abundance of each species the same as observed.

**Figure S4.** The examples of the interspecific spatial pattern (bivariate point process) of three types of matched species pairs (dominating species vs. dominating species, dominating species vs. common species and dominating species vs. rare species) in the fenced and grazed plots. The solid lines are the pair-correlation functions and the two dotted lines represent the upper and the lower limits of the 95% confidence envelopes of the 99 Monte Carlo simulations respectively. Patterns of intraspecific association across spatial scales based on the function *g*_12_(*r*). Associations were considered significantly non-random if they fell outside the 95% confidence intervals of the null model (see Material and Methods).
