# Supplementary material for: Grazer exclusion alters plant spatial organization at multiple scales, increasing diversity
Source: Ecol Evol. 2013 Sep 2;3(10):3604–12. doi: 10.1002/ece3.743 (PMC3797503; doi:10.1002/ece3.743)

**Supporting information**

**Table S1.** The average content of soil and topographic variables and the standard deviations (in brackets) in the fenced and grazed plots of site 1 and site 2.

|  |  | N  (mg/Kg) | P  (mg/Kg) | PH | Moisture  (mg/Kg) | Fe  (mg/Kg) | Mg  (mg/Kg) | Ca  (mg/Kg) | | Na  (mg/Kg) | | K  (mg/Kg) | C  (mg/Kg) | | slope |
| --- | --- | --- | --- | --- | --- | --- | --- | --- | --- | --- | --- | --- | --- | --- | --- |
| Site 1 | Fenced | 26.61(1.64) | 39.80(0.39) | 7.93(0.27) | 81510(5614.61) | 34.84(1.59) | 11.13(0.50) | 35.35(6.32) | | 13.04(0.83) | | 21.48(1.12) | 28.99(2.12) | | 16.12(5.45) |
|  | Grazed | 28(2.24) | 57.62(1.47) | 7.93(0.18) | 90630(6879.20) | 35.40(1.21) | 10.80(0.36) | 32.52(5.82) | | 12.39(0.77) | | 21.34(0.93) | 30.11(2.13) | | 16.12(5.48) |
| Site 2 | Fenced | 15.51(3.08) | 80.82(3.51) | 7.98(0.11) | 177861(5680.01) | 31.58(0.91) | 12.01(0.28) | 33.07(7.97) | 12.48(0.43) | | 215(0.51) | | | 68.24(1.83) | 5.68(4.95) |
|  | Grazed | 21.85(3.05) | 88.68(2.35) | 8.17(0.07) | 179590(4557.51) | 33.20(1.81) | 12.07(0.37) | 18.20(7.56) | 12.39(0.48) | | 213(0.46) | | | 71.95(1.93) | 6.78(5.12) |

**Table S2.** Species composition in the 10 m*×*10 m plots of the fenced and grazed areas of two meadows. The cross indicates that the species was detected in the plot.

|  | site1 | | site2 | |
| --- | --- | --- | --- | --- |
| species | Fenced | Grazed | Fenced | Grazed |
| *Scirpus pumilus* | *×* | *×* | *×* | *×* |
| *Thalictrum alpinum L* | *×* | *×* | *×* | *×* |
| *Carex kansuensis* | *×* | *×* | *×* | *×* |
| *Kobresia humilis* | *×* | *×* | *×* | *×* |
| *Anemone obtusiloba* | *×* | *×* | *×* | *×* |
| *Anemone rivularis* | *×* | *×* | *×* | *×* |
| *Delphinium kamaonense* | *×* | *×* | *×* | *×* |
| *Potentilla anserina* | *×* | *×* | *×* | *×* |
| *Potentilla fragarioides* | *×* | *×* | *×* | *×* |
| *Saussurea nigrescens* | *×* | *×* | *×* | *×* |
| *Saussurea hieracioides* | *×* | *×* | *×* | *×* |
| *Taraxacum maurocarpum* | *×* | *×* | *×* | *×* |
| *Euphorbia altotibetica* | *×* | *×* | *×* | *×* |
| *Geranium pylzowianum* | *×* | *×* | *×* | *×* |
| *Gentiana straminea* | *×* | *×* | *×* | *×* |
| *Swertia tetraptera* | *×* | *×* | *×* | *×* |
| *Anaphalis lactea* | *×* | *×* | *×* | *×* |
| *Leontopodium nanum* | *×* | *×* | *×* | *×* |
| *Radix Bupleuri* | *×* | *×* | *×* | *×* |
| *Oxytropis ochrocephala* | *×* | *×* | *×* | *×* |
| *Agrostis gigantea* | *×* | *×* | *×* | *×* |
| *Lancea tibetica* | *×* | *×* | *×* | *×* |
| *Plantago depressa* | *×* | *×* | *×* | *×* |
| *Polygonum viviparum* | *×* | *×* | *×* | *×* |
| *Gentiana sino-ornata* | *×* | *×* | *×* |  |
| *Elymus nutans* | *×* | *×* | *×* | *×* |
| *Poa pratensis* | *×* | *×* | *×* | *×* |
| *Pedicularis szetschuanica* | *×* | *×* | *×* | *×* |
| *Agropyron cristatum* | *×* | *×* | *×* | *×* |
| *Cirsium setosum* | *×* | *×* | *×* | *×* |
| *Leymus secalinus* | *×* | *×* | *×* | *×* |
| *Viola tuberifera* | *×* | *×* | *×* | *×* |
| *Ajuga ovalifolia* | *×* | *×* | *×* | *×* |
| *Tibetia himalaica* | *×* | *×* | *×* | *×* |
| *Vicia multicaulis* | *×* | *×* | *×* |  |
| *Stipa aliena* | *×* | *×* | *×* | *×* |
| *Salvia roborowskii* | *×* | *×* | *×* | *×* |
| *Rumex patientia* | *×* | *×* |  |  |
| *Euphrasia regelii* | *×* | *×* | *×* | *×* |
| *Potentilla potaninii* | *×* | *×* | *×* | *×* |
| *Koeleria cristata* | *×* | *×* | *×* | *×* |
| *sphaerostachyum Meissn* | *×* | *×* | *×* |  |
| *Halenia elliptica* | *×* | *×* |  |  |
| *Artemisia tangutica* | *×* | *×* | *×* | *×* |
| *Cerastium fontanum* | *×* | *×* |  | *×* |
| *Melandrium himalayense* | *×* | *×* |  |  |
| *Cnidium monnieri* | *×* | *×* |  | *×* |
| *CompositaeAjaniatenuifolia* | *×* | *×* | *×* |  |
| *Anaphalis hancockii* | *×* | *×* | *×* | *×* |
| *Desterorum Spreng* | *×* | *×* | *×* | *×* |
| *Daucus carota L* | *×* | *×* | *×* | *×* |
| *Equisetum arvense L* | *×* | *×* | *×* | *×* |
| *Arenaria serpyllifolia L* | *×* | *×* | *×* | *×* |
| *Galium verum L* | *×* | *×* | *×* |  |
| *Saussurea parviflora (Poir.) DC* | *×* | *×* | *×* | *×* |
| *Medicago sativa Linn* | *×* | *×* | *×* | *×* |
| *Stellera chamaejasme* | *×* | *×* | *×* | *×* |
| *Iris lactea Pall. var* | *×* | *×* | *×* | *×* |
| *Potentilla bifurca L* |  | *×* | *×* | *×* |
| *Agrostis trinii* |  | *×* | *×* | *×* |
| *Melandrium himalayense* | *×* |  | *×* |  |
| *Hispidus (Thunb. ) Less* | *×* |  | *×* |  |
| *Thernopsis alicentiana* | *×* |  | *×* |  |
| *Herminium monorchis* | *×* |  | *×* | *×* |
| *Parnassia trinervis* | *×* |  | *×* | *×* |
| *Saposhnikoviadivaricata* | *×* |  | *×* |  |
| *Astragalus polycladus* | *×* |  | *×* |  |
| *Sphallerocarpus gracilis* | *×* |  | *×* |  |
| *Artemisia stracheyi* | *×* |  | *×* | *×* |
| *Sonchus transcaspicus* | *×* |  | *×* | *×* |
| *Taraxacum mongolicum* | *×* |  |  | *×* |
| *Hippophae rhamnoides* | *×* |  | *×* | *×* |
| *Roegneria nutans* | *×* |  | *×* |  |
| *Allium sikkimense* | *×* |  | *×* |  |
| *Ligularia sagitta* | *×* |  | *×* |  |
| *Saussurea pachyneura* | *×* |  | *×* | *×* |
| *Deschampsia caespitosa* | *×* |  |  |  |
| *Lomatogonium macranthum* | *×* |  | *×* | *×* |
| *Melandrium himalayense* | *×* |  | *×* |  |
| *Anemone trullifolia* | *×* |  | *×* |  |
| *Gentianopsis paludosa* | *×* |  | *×* | *×* |
| *Trollius farreri* | *×* |  | *×* |  |
| *Veronica eriogyne* | *×* |  | *×* | *×* |
| *Capsella bursa-pastoris* | *×* |  | *×* |  |
| *Scirpus pumilus* | *×* |  | *×* |  |
| *Aconitum carmichaeli Debx* |  |  |  | *×* |
| *Aster alpinus L* |  |  | *×* |  |
| *Poa pachyantha Keng* |  |  | *×* |  |
| *Bromus staintonii Meld* |  |  | *×* |  |
| *Gentiana squarrosa Ledeb.* |  |  | *×* | *×* |

**Figure S1.** Changes in resource heterogeneity as a function of distance between sampling points in site 1. Each point represents the average coefficient of variation for all pairwise points measured at that distance, with filled circles representing the CV in grazed areas and open circles representing the CV in fenced plots. Labels above each panel indicate the soil resource in site 1.

**
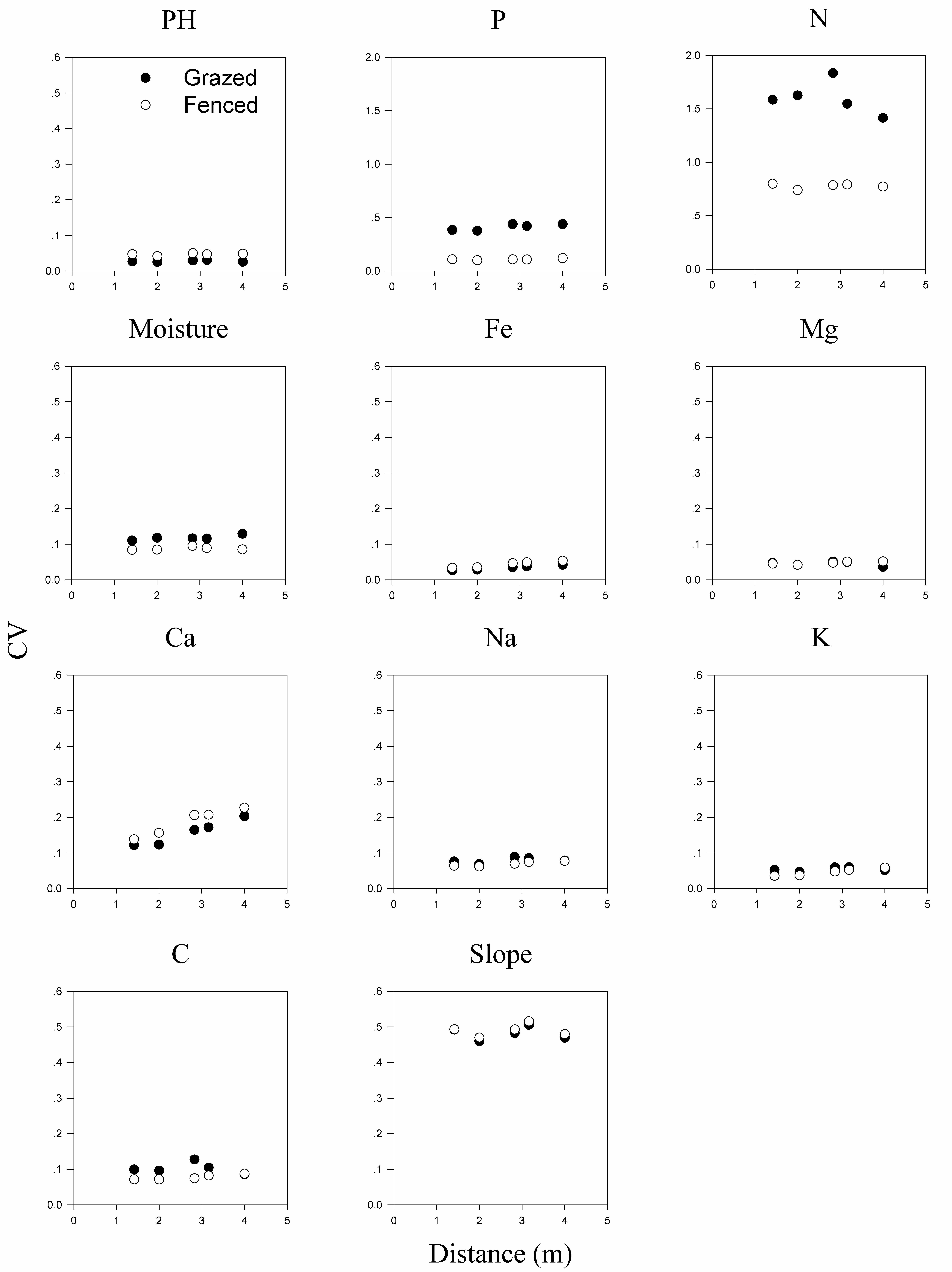
**

**Figure S2.** Changes in resource heterogeneity as a function of distance between sampling points in site 2. Each point represents the average coefficient of variation for all pairwise points measured at that distance, with filled circles representing the CV in grazed areas and open circles representing the CV in fenced plots. Labels above each panel indicate the soil resource in site 2.


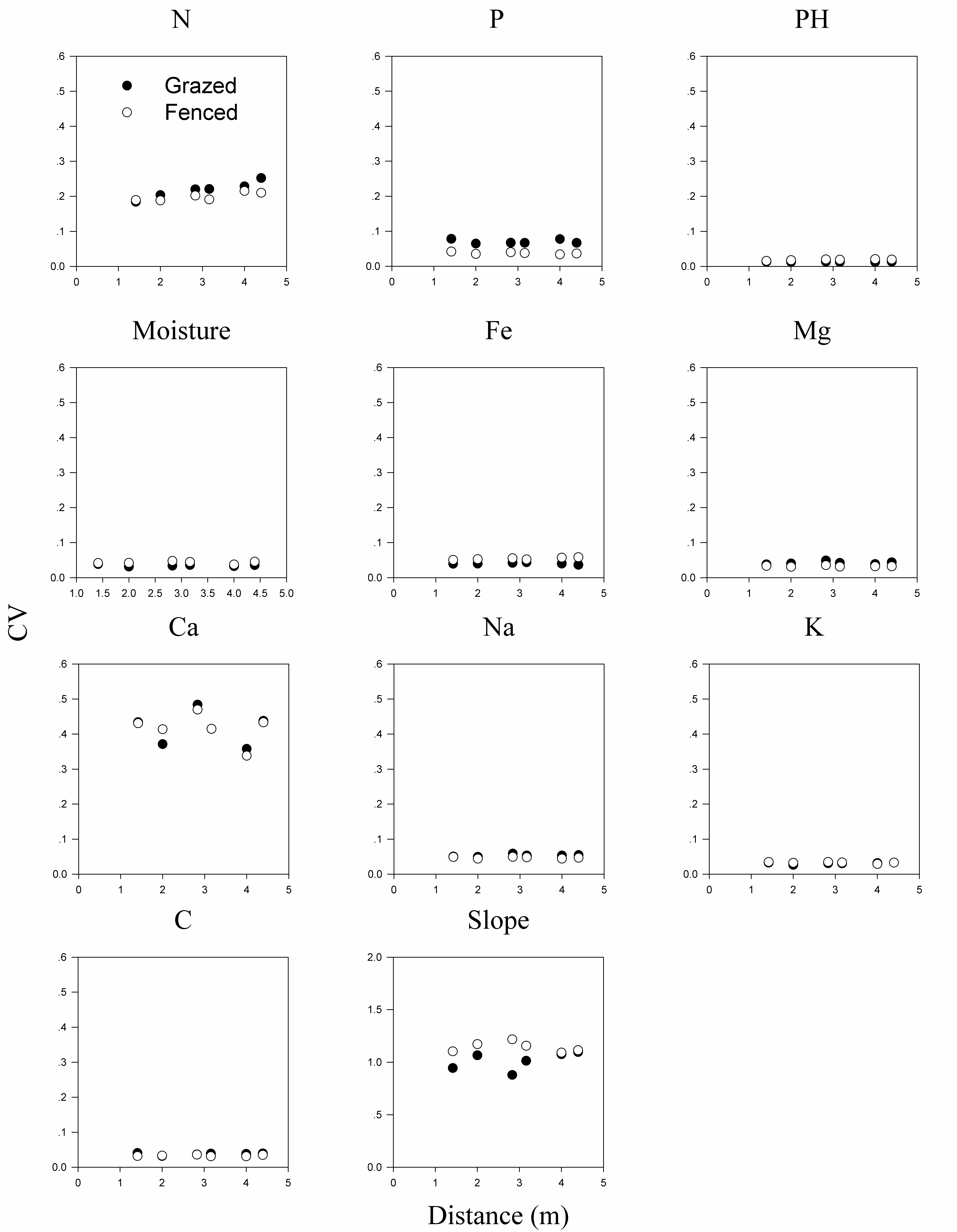


**Figure S3.** The examples of the intra-specific spatial patterns (univariate point process) of the three types of species (dominating, common and rare species) in the fenced and grazed plots. The solid lines are for the values of g function and the two dotted lines represent the upper and the lower limits of the 95% confidence envelopes of the 99 Monte Carlo simulations by randomly labeling all the aboveground-ramets in the plot while keeping the abundance of each species the same as observed.


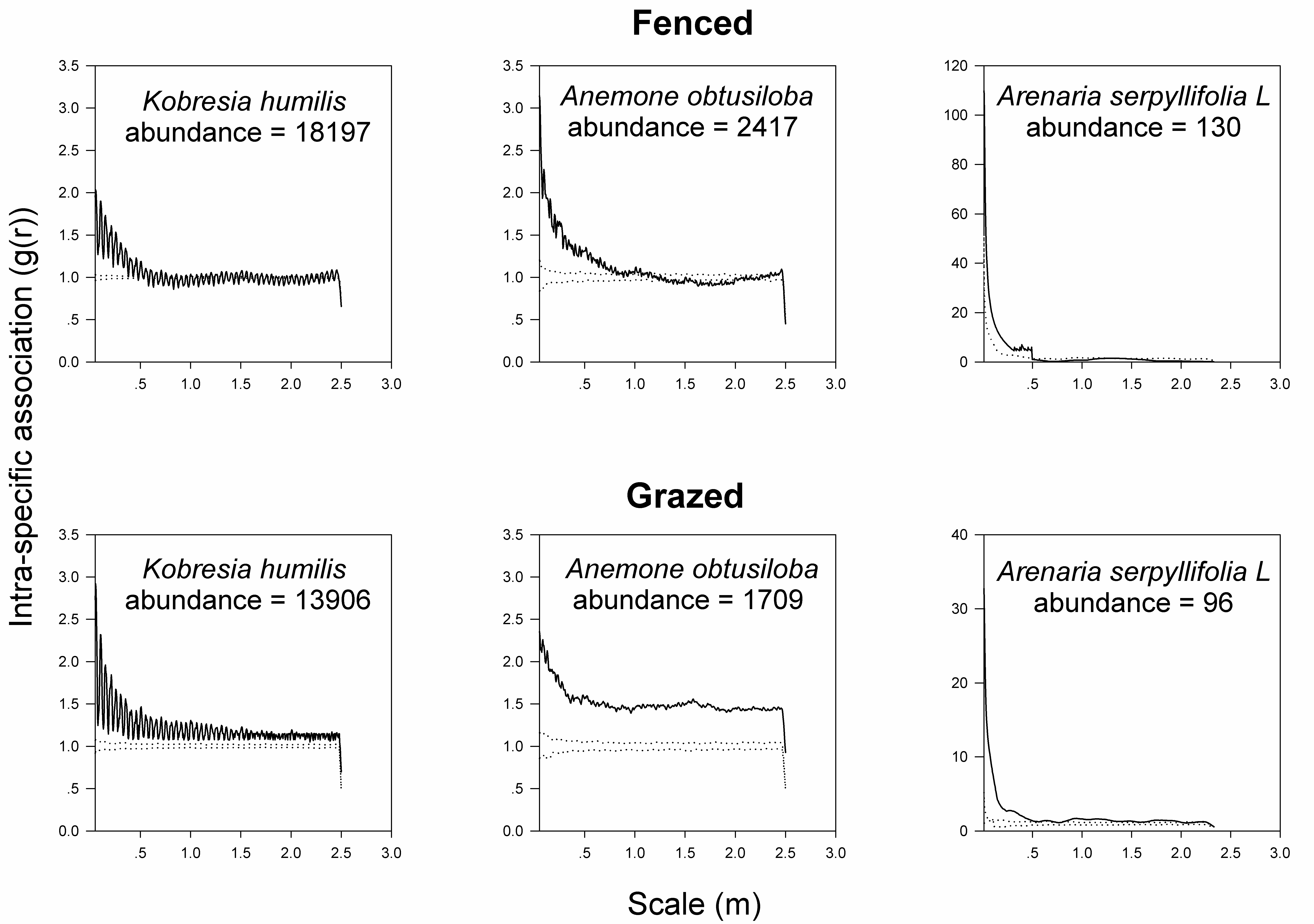


**Figure S4.** The examples of the inter-specific spatial pattern (bivariate point process) of three types of matched species pairs (dominating species vs. dominating species, dominating species vs. common species and dominating species vs. rare species) in the fenced and grazed plots. The solid lines are the pair-correlation functions and the two dotted lines represent the upper and the lower limits of the 95% confidence envelopes of the 99 Monte Carlo simulations respectively. Patterns of intraspecific association across spatial scales based on the function g_12_(r). Associations were considered significantly non-random if they fell outside the 95% confidence intervals of the null model (see methods).


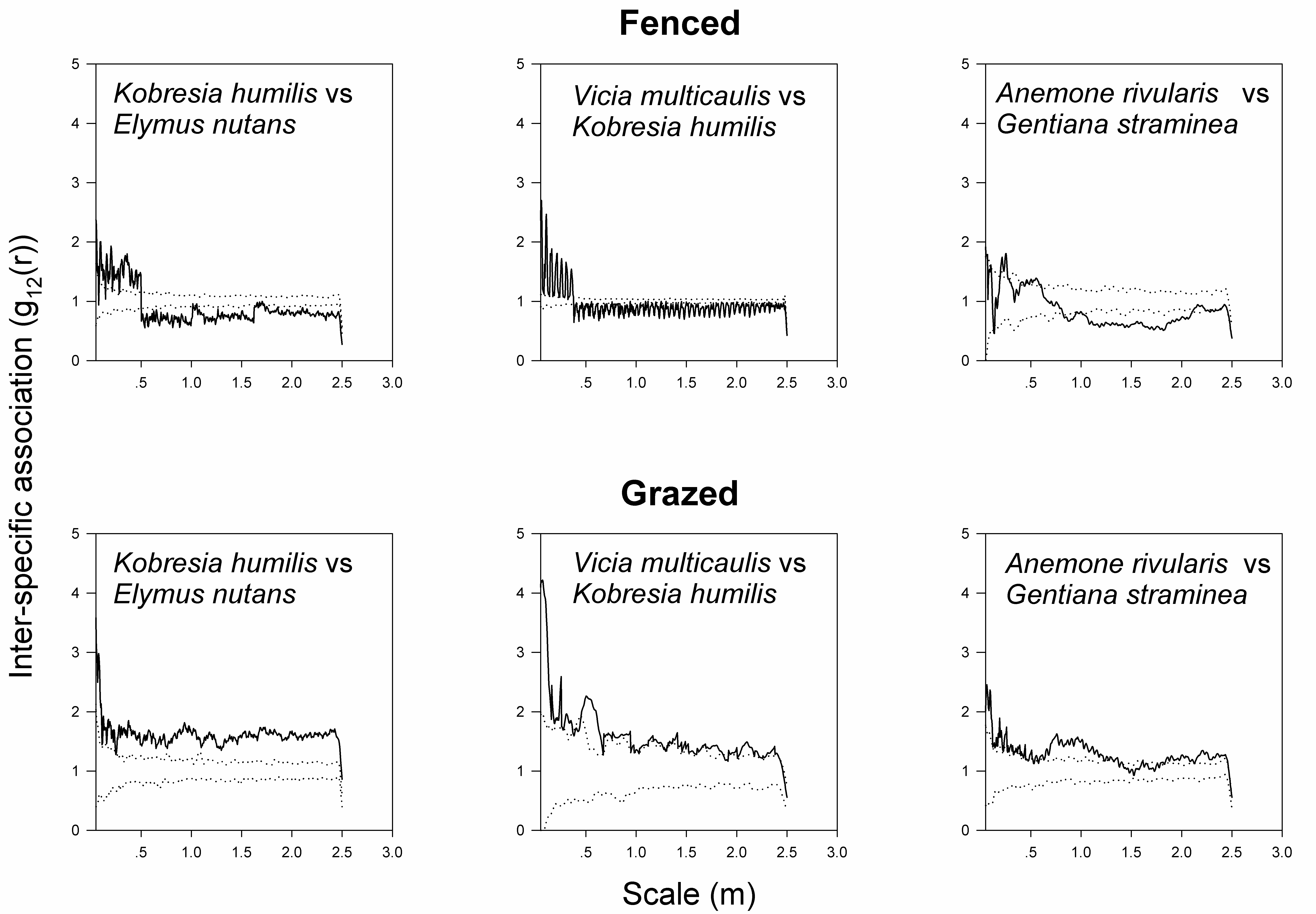

Supplement: Supplementary file 1 [file ece30003-3604-SD1.doc]
